# Choriocapillaris Flow-Enriched Prediction of Retinal Sensitivity Using OCT-Derived Biomarkers in Intermediate Age-Related Macular Degeneration

**DOI:** 10.3390/jcm15093392

**Published:** 2026-04-29

**Authors:** Johannes Schrittwieser, Lukas Kuchernig, Virginia Mares, Irene Steiner, Klaudia Birner, Florian Frommlet, Enrico Borrelli, Hrvoje Bogunović, Stefan Sacu, Gregor S. Reiter

**Affiliations:** 1 Department of Ophthalmology and Optometry, Medical University of Vienna, 1090 Vienna, Austria; 2 Medical University of Vienna, Center for Medical Data Science, Institute of Medical Statistics, 1090 Vienna, Austria; 3 Department of Surgical Sciences, University of Turin, 10124 Turin, Italy; 4 Department of Ophthalmology, “City of Health and Science” Hospital, 10126 Turin, Italy; 5 Institute of Artificial Intelligence, Center for Medical Data Science, Medical University of Vienna, 1090 Vienna, Austria

**Keywords:** microperimetry, retinal sensitivity, intermediate age-related macular degeneration, OCT, choriocapillaris, OCT angiography, flow-deficits, deep-learning, artificial intelligence

## Abstract

**Objectives**: To assess the association of structural biomarkers derived from optical coherence tomography (OCT) and choriocapillaris (CC) flow information with point-wise retinal sensitivity (PWS) measured by microperimetry (MP) in intermediate age-related macular degeneration (iAMD). **Methods:** Patients with iAMD received imaging with spectral-domain (SD)-OCT (Spectralis, Heidelberg Engineering) and OCT-angiography (OCT-A) (PLEX Elite 9000, ZEISS). In addition, MP examinations in photopic setting (MP-3, NIDEK) and mesopic background illumination (MAIA2, ICare) were performed. The thickness of the ellipsoid-zone (EZ) and the outer nuclear layer (ONL), as well as the volume of drusen and HRF, were segmented using deep-learning (DL)-based approaches. CC flow deficit percentage (FD%) was extracted from OCT-A slabs using a novel binarization method. Semiautomatic co-registration of MP examinations, OCT-A slabs, and OCT volumes was performed. Three exploratory models were calculated using multivariable mixed-effects models: (1) structure–function (SF) using structural OCT biomarkers, (2) flow–function (FF) utilizing OCT-A derived flow information, and (3) structure–flow–function (SFF) incorporating both OCT and OCT-A data. Model performance was evaluated using AIC and BIC criterion. **Results**: 19 eyes of 19 patients were evaluated, totalling 3297 MP-stimuli, 1873 B-scans, and 19 OCT-A slabs. Mean (SD) age was 76 (7) years, and sensitivity was 26.0 (3.36) dB in the MP-3 and 22.42 (3.64) dB in the MAIA2. Mesopic MAIA2 examinations showed significantly lower PWS values (−3.56 to −3.63 dB; *p* < 0.001). Drusen and HRF volume decreased PWS (−0.6 [95% CI: −1.04; −0.16] dB/nL; *p* = 0.007 and −9.56 [95% CI: −12.86; −6.26] dB/nL; *p* < 0.001), while ONL was positively associated with PWS (0.06 [0.05; 0.07] at an eccentricity of 5.2°; *p* < 0.001) in the SF model. CC FD% was not significantly associated with PWS in the FF and the SFF model (*p* > 0.05 in both cases). In the SFF model drusen volume (−1.69 [95% CI: −2.09; −1.29] dB/nL; *p* < 0.001), EZ (0.04 [95% CI: 0.02; 0.06] dB/µm; *p* < 0.001), and ONL thickness (0.03 [95% CI: 0.02; 0.04] dB/µm; *p* < 0.001) were significant predictors for PWS. The SF model exhibited the lowest AIC and BIC indicating best model performance. **Conclusions:** Structural parameters derived from SD-OCT such as HRF, drusen volume, and outer retinal layer thickness may be more closely associated with PWS, with CC FD% as an OCT-A-derived metric contributing limited additional explanatory benefit in cross-sectional analyses.

## 1. Introduction

Structure–function analyses are of substantial value in retinal research, especially in age-related macular degeneration (AMD), as visual impairment often results from complex interactions between different altered retinal structures, ultimately causing functional loss [1]. These structure–function associations have been extensively studied in this disease [2]. AMD is influenced by environmental and genetic factors and is one of the leading causes of visual impairment around the globe [3]. AMD is characterized by the development of drusen, pigmentary abnormalities, atrophy, and macular edema visible on fundoscopy or color fundus photography [4]. In addition, three-dimensional OCT examination reveals biomarkers such as hyperreflective foci (HRF), intraretinal and subretinal fluid (IRF, SRF), pigment epithelium detachment, ellipsoid zone (EZ), and retinal pigment epithelium (RPE) loss [3]. These biomarkers were shown to have an influence on visual function, such as best-corrected visual acuity (BCVA) [5], low-luminance visual acuity (LLVA) [6], and retinal sensitivity (RS) measured by microperimetry (MP) [7,8,9].

MP, also called fundus-controlled perimetry, is an especially valuable functional testing modality for structure–function analyses, as it enables comprehensive assessment of RS by projecting multiple stimuli onto predefined areas of the retina, correcting for eye movements, and testing fixation stability [10,11]. Derived sensitivity maps are especially useful for the detection of subclinical functional changes outside of the foveal center, which might not be captured by other functional tests [10]. By co-registering MP examinations with structural OCT volumes, the point-wise impact of structural biomarkers on retinal function can be precisely investigated [7,8,9]. Previous studies already showed that RS measured by MP is significantly lower in patients with all AMD stages compared to healthy controls [12]. MP is also established in other retinal diseases such as diabetic retinopathy [13].

However, there is a growing body of evidence that not only structural changes visible on OCT and CFP, but also alteration in the microvasculature of the choriocapillaris (CC), are associated with progression of the disease [14]. Histopathologic examinations revealed an increasing number of non-perfused capillaries (ghost vessels) underlying drusen, leading to hypoxia and cell damage in the outer retinal cells and the RPE [15]. These changes in the CC can be visualized with OCT-angiography (OCT-A) as CC flow deficits (FD) [16,17,18]. It is hypothesized that these alterations in the microvasculature favor the formation of structural alterations such as drusen [19,20,21], subretinal drusenoid deposits (SDD) [22], RPE attenuation [23], and photoreceptor degeneration visible on the structural OCT as EZ thinning [24]. These changes drive progression towards late-stage AMD with development of complete RPE and outer retinal atrophy (cRORA) [25] and/or macular neovascularization (MNV) [26,27], indicating a strong vascular component in the development and progression of the disease. While the loss of the CC is a hallmark of late atrophic AMD [14] there is also evidence that loss in the microvasculature is also occurring in early and intermediate stages of the disease, preceding structural signs of late-stage AMD [24].

Therefore, relying solely on structural alterations from the OCT might not fully capture all relevant aspects of functional impairment, especially when evaluating subtle signs of disease progression in early and intermediate stages. This study presents a novel approach integrating point-wise MP with spatially co-registered OCT- and OCT-A derived biomarkers, enabling direct comparison of structure-only, flow-only, and combined structure–flow models within the same retinal locations.

The purpose of this study is to investigate the association between OCT-derived structural biomarkers and OCT-A-derived CC FD on RS measured by MP in iAMD and to compare the predictive value of three exploratory models.

## 2. Materials and Methods

### 2.1. Study Population

Patients aged 50 years or older were recruited at the Department of Ophthalmology and Optometry, Medical University of Vienna, Austria. All participants provided written informed consent before any study procedures. The study adhered to the tenets of the Declaration of Helsinki and was approved by the Ethics Committee of the Medical University of Vienna (EK: 1399/2021).

The definition of early to intermediate age-related macular degeneration (AMD) followed the criteria proposed by Ferris et al. [28]. Exclusion criteria included any signs of late AMD, such as cRORA, or evidence of MNV according to the consensus-based definitions [25,26]. Additional exclusion criteria included a history of glaucoma, significant media opacities, amblyopia, and refractive errors greater than ± 5 diopters. Only one eye per participant was included in the analysis. When both eyes met the eligibility criteria, the eye with superior OCT image quality was selected, as previously described [8]. This cohort has been described previously in Birner et al. [8].

### 2.2. Imaging Protocol

Spectral-domain OCT (SD-OCT) imaging was performed using the Spectralis HRA + OCT (Heidelberg Engineering, Heidelberg, Germany). Each volume covered a 20° × 20° macular field and comprised 97 high-resolution B-scans (1024 A-scans per B-scan) acquired with an automatic real-time (ART) averaging value of 16 frames, centered on the fovea. OCT-A imaging was acquired using the PLEX Elite 9000 (Carl Zeiss Meditec, Dublin, CA, USA), operating at 200 kHz and covering the central 6 mm × 6 mm region. OCT-A volumes were required to have a signal strength ≥ 7 and be free of significant artifacts (e.g., motion or blink artifacts) to be included in the analysis. The CC slab was defined as a 20 µm thick layer starting at the outer boundary of Bruch’s membrane (BM), corresponding to the PLEX Elite 9000 default setting. This definition approximates histological CC thickness while accommodating anatomical variability and the axial resolution of OCT imaging [18]. Each scan was reviewed to verify correct BM segmentation.

### 2.3. Testing Protocol

The testing protocol has been described in detail in Coulibaly et al. [29]. In brief, at the same visit, two successive MP examinations were performed using both the MP-3 (NIDEK Co., Ltd., Gamagori, Japan) and MAIA2 (iCare S.p.A., Padova, Italy) devices, resulting in a total of four examinations per participant. The order of device acquisition was randomized, and a 10-min rest interval was provided between sessions. Each MP test used an identical fovea-centered grid consisting of 45 Goldmann III stimuli, following a 4-2 staircase thresholding strategy. The MAIA2 was operated under mesopic conditions (background luminance: 4 asb; 1.27 cd/m^2^), while the MP-3 was performed under photopic conditions (background luminance: 31.4 asb; 10 cd/m^2^).

### 2.4. Multimodal Image Registration

**Figure 1:**
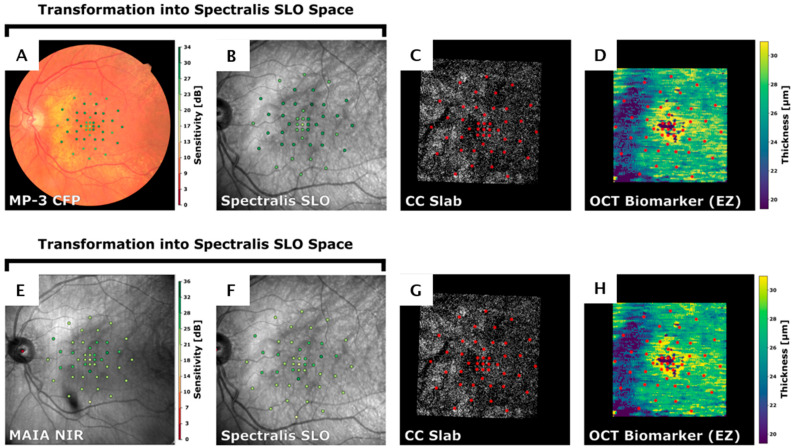
Multimodal registration of microperimetry sensitivity maps with structural and flow biomarkers. (**A**,**E**) Mesopic microperimetry (MP) (MAIA2 NIR)/photopic MP (MP-3 CFP) map showing stimulus locations color-coded by retinal sensitivity (dB). (**B**,**F**) The same stimuli registered to the Spectralis SLO reference frame. Colored circles represent individual microperimetry stimulus locations, with circle color corresponding to the measured sensitivity value at that location. (**C**,**G**) Corresponding locations (red circles) overlaid on the PLEX Elite 9000 choriocapillaris (CC) slab, used for local quantification of flow deficits. (**D**,**H**) Overlay of the same coordinates on an OCT-derived ellipsoid zone (EZ) thickness map, obtained from validated deep learning-based layer segmentation. Each stimulus location was analyzed within a 140 µm circular region of interest for localized structure–flow–function correlations.

For multimodal registration, vessel masks were first extracted from each image type (Spectralis scanning laser ophthalmoscopy [SLO], MAIA2 near-infrared reflectance [NIR], MP-3 color fundus photograph [CFP], and PLEX Elite 9000 OCT-A retina slab) using the SLOctolyzer package [30], which applies a deep learning-based vessel binarization approach. Subsequently, the Scale-Invariant Feature Transform (SIFT) algorithm was used to automatically detect corresponding keypoints between the moving (MAIA2 NIR, MP-3 CFP, or OCT-A) and fixed (Spectralis SLO) images. When automatic keypoint matching failed or was insufficient, corresponding landmark pairs were manually selected to ensure accurate alignment. An affine transformation was then computed from the matched keypoints and applied to register the moving image to the Spectralis SLO reference frame. The resulting transformation matrix was stored and subsequently applied to map all associated coordinate data (e.g., MP stimulus locations and OCT-A CC slab) into the Spectralis SLO coordinate system. Registration accuracy was visually inspected for each image pair using vessel overlays and point correspondences. Only image pairs demonstrating accurate vessel alignment across modalities were accepted for further analysis. Accuracy of our image registration pipeline was shown previously [31] (Figure 1).

### 2.5. Data Analysis Pipeline

For MAIA2 devices, stimulus locations were converted from degrees to pixels using the report-specific pix2deg ratio. For MP-3 devices, per-stimulus RGB pixel offsets were used directly. Absolute stimulus coordinates were computed within the native fundus frame. Using the previously stored transformation parameters, all stimulus coordinates were transformed into the Spectralis SLO reference frame.

From the SLO pixel spacings, anisotropic in-plane OCT spacings were derived, allowing precise mapping between SLO and OCT pixel coordinates. Around each transformed stimulus, a single circular region of interest (ROI) with a 140 µm diameter was analyzed. ROIs were included only if fully contained within the analysis mask (i.e., regions where OCT and OCT-A data were both available) to prevent edge effects and invalid-pixel bias.

### 2.6. Structural Biomarkers

**Figure 2:**
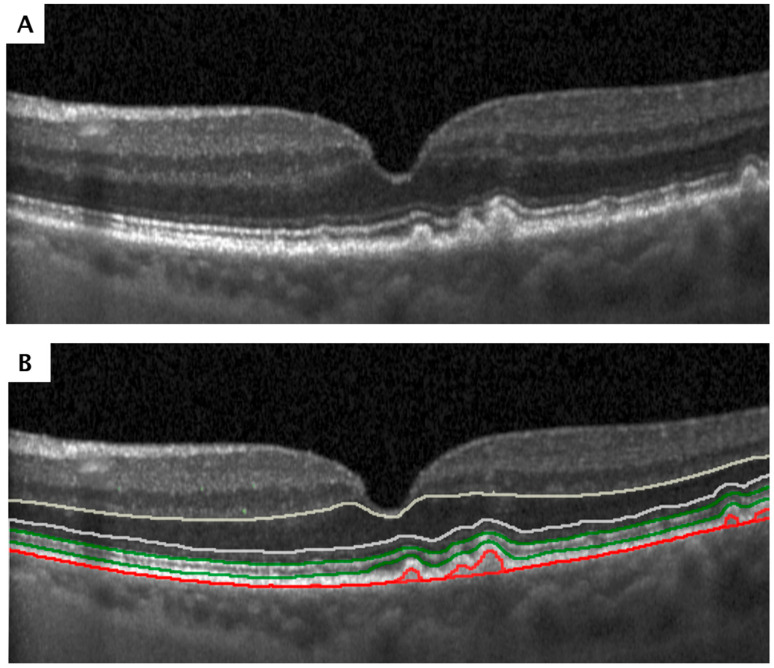
(**A**) Optical coherence tomography (OCT) of intermediate age-related macular degeneration (iAMD). (**B**) Deep-learning (DL)-based segmentation of disease-specific biomarkers. Outer nuclear layer (ONL) thickness is shown in white, ellipsoid-zone (EZ) thickness in green, and drusen volume in red. The colored segmentation lines indicate the retinal layer boundaries used to quantify each biomarker: white lines delineate the ONL, green lines delineate the EZ, and red lines outline drusen volume.

EZ thickness, defined as the distance between the inner boundary of the EZ and the outer boundary of the interdigitation zone (IZ), was quantified through automated layer segmentation using a validated deep learning algorithm (Figure 2) [32,33]. The algorithm for automatic EZ segmentation used a deep learning–based approach built on U-shaped fully convolutional neural networks. A total of 40 OCT datasets from 40 patients were included (16 with diabetic macular edema and 24 with retinal vein occlusion), and the data were divided into training (25 volumes), validation (three volumes), and test (12 volumes) sets [32]. The ONL was defined as the distance between the outer boundary of the outer plexiform layer (OPL) and the inner boundary of the external limiting membrane (ELM) [34]. To ensure optimal precision in ONL thickness measurements, deep learning-based segmentations of the OPL outer boundary were manually corrected when necessary, and the Henle fiber layer was included within the ONL thickness definition. ELM annotations were also manually annotated. Hyperreflective foci (HRF) were identified using a fully automated convolutional neural network-based segmentation pipeline, as previously described [35]. For automated HRF quantification, a deep-learning method built on a convolutional neural network with an encoder–decoder design was applied. A total of 145 OCT volumes, including 60 from AMD, 42 from RVO, and 43 from DME cases, were included for model development and evaluation. These data were then divided into separate subsets comprising 119 OCT scans (1051 images) for training, six OCT scans (41 images) for validation, and 20 OCT scans (137 images) for testing [35].

The outer boundary of the retinal pigment epithelium (OB-RPE) and Bruch’s membrane (BM) were segmented using the Iowa Reference Algorithm with modified smoothness constraints, as previously reported [36]. Drusen volume was calculated by summing all voxels in which the OB-RPE was elevated ≥ 8 μm above the BM and multiplying the voxel count by the voxel volume [36]. All manual annotations were performed by a single trained expert reader. In cases of uncertainty, the readers discussed the findings with a retinal specialist (G.S.R.) until a consensus was reached.

### 2.7. Choriocapillaris Flow Deficit Percentage (FD%)

The CC binarization method was developed by our healthy controls group and validated in patients affected by GA [31]. In brief, prior to binarization, a compensation method described by Zhang et al. [37] was applied to correct for masking and unmasking artifacts. In addition, brightness differences were compensated using histogram normalization.

Histological studies of healthy eyes showed that 79% of the CC area consists of vasculature, physiologically resulting in 21% of the area exhibiting no vessel tissue [38,39]. Therefore, in accordance with these histopathological findings, we determined a binarization threshold in a cohort of healthy eyes resulting in 21% of the pixels being classified as flow deficits. This resulted in a threshold of 47 grey levels with a dynamic range of 0–255 [31].

Artifact-compensated and normalized CC en face slabs were binarized using the above-mentioned intensity threshold, with white pixels indicating flow deficits. FD% was calculated as ratio (white (no flow)/valid (flow) × 100) for each ROI (140 µm diameter) representing a MP stimulus. Although most studies visualize flow deficits in black corresponding to the OCT-A CC slab, we chose to use the color white instead because of better contrast and visualization. All analyses were performed in Python (version 3.11.13).

### 2.8. Statistical Analysis

For approximately normally distributed variables mean ± standard deviation was calculated. Skewed variables were reported with median and Q1-Q3. All analyses were conducted with the subset of complete observations in EZ-thickness, ONL-thickness, drusen volume, HRF-volume, and FD% (3297 stimuli, 19 patients). Three different multivariable mixed-effects models with PWS as dependent variable and patient as random intercept were calculated for this exploratory analysis (R-package nlme 3.1–168, R-function lme) [40]: (1) a structure–function (SF) model that assesses the association of structural OCT-biomarkers with PWS. The following parameters were included according to Birner et al. [8]: device type (MAIA2; MP-3), EZ-thickness (µm), ONL-thickness (µm), drusen volume (nL), HRF volume (nL; HRF values < 0.06 nl were set to 0 to rule out eventual segmentation errors), and eccentricity (°). Interaction terms between eccentricity and EZ-thickness and eccentricity and ONL-thickness were included. To facilitate the interpretation of interaction terms, ONL and EZ-thickness were mean-centered, and eccentricity was centered around 5.2°; (2) a flow–function (FF) model analyzing the effect of OCT-A derived FD% on PWS was created. Fixed factors included: device type, age (years), eccentricity (°), and FD%; (3) in the structure–flow–function (SFF) model the effect of structural information derived from OCT in combination with flow information from OCT-A on PWS was assessed. Fixed factors were device type, age (years), eccentricity (°), drusen volume (nL), EZ-thickness (µm), ONL-thickness (µm), and FD%. The interaction between drusen volume and FD% was also analyzed, but not included in the model to improve interpretability, as the interaction term was not statistically significant. Variables of model 2 and 3 were selected based on theoretical considerations. The explanatory capabilities of the three models were then compared using the Akaike information criterion (AIC) and the Bayesian information criterion (BIC) using maximum likelihood (ML) estimation. Estimates and *p*-values, as well as the marginal R^2^ (R^2^m; R-function r.squaredGLMM, R-package MuMIn_1.48.11) [41] and intraclass correlation coefficients (ICCs; calculated by dividing the random effect variance by the sum of the random effect variance and the residual variance) were obtained from models using restricted maximum likelihood (REML). The variance inflation factor (VIF) was calculated for signs of multicollinearity using the R-function check_collinearity (R-package performance_0.15.2) [42] (Supplement Table S1). Correlations between independent variables were analyzed by Spearman correlation coefficients (rs). Furthermore, residual vs. fitted plots were created (Supplement Figures S1–S3). All statistical analyses were carried out with R 4.5.1. The analyses are hypothesis-generating and hence the interpretation of the *p* values are exploratory.

## 3. Results

Twenty eyes of 20 patients with iAMD were included in this study. Descriptive statistics of PWS and SD-OCT parameters have been published previously in Birner et al. [8]. However, no OCT-A data was analyzed in the aforementioned study.

In the current analyses one eye was excluded, because flow–function was not available. Furthermore, the analyses were conducted with the subset of stimuli with complete OCT-A and SD-OCT measurements. While PWS measurements were available in 3420 stimuli (180 stimuli per eye), thickness and volumes were measured in 3367 stimuli. For OCT-A, measurements in 3338 stimuli were available, 3297 of which were stimuli with both OCT-A and SD-OCT measurements. Results refer to the subset with complete observations. Mean (SD) age of the 19 patients was 76 (7) years. Mean PWS in the MP-3 was 25.88 (3.32) dB and 26.12 (3.39) dB for the first and the second run, respectively, while PWS in the MAIA2 was 22.49 (3.65) dB in the first examination and 22.35 (3.63) dB. Mean flow-deficit percentage in the MP-3 was 25.30 (12.30) % and 25.27 (12.36) % for the first and the second run, respectively, while in the MAIA2 flow-deficit percentage was 26.19 (12.71) % in the first examination and 25.42 (12.15) % in the second. Descriptive statistics are summarized in Table 1. For drusen volume and HRF-volume, the descriptive statistics refer to measurements with value > 0. Drusen volume > 0 was measured in 1409/3297 (42.7%) stimuli, and HRF volume > 0 was measured in 124/3297 (3.8%) stimuli.

### 3.1. Correlation of Independent Parameters

Drusen volume showed a negative correlation with EZ-thickness (rs = −0.35) and eccentricity (rs = −0.45). EZ thickness was positively associated with eccentricity (rs = 0.24) and ONL-thickness (rs = 0.09). ONL was moderately negatively correlated with eccentricity (rs = −0.67). To check for multicollinearity the VIF values ranged from 1.0–3.76 in the FF and SFF model and from 1.02 to 4.92 in the SF model; therefore, no signs of collinearity could be observed, and all parameters could be included in the models (Table 2, Supplement Table S1).

### 3.2. Association of Biomarkers and PWS

MAIA2 examinations in a mesopic setting showed lower PWS compared to MP-3 examinations (estimates ranging from −3.56 to −3.63 dB; *p* < 0.001) compared to the MP-3 with a photopic background illumination.

In the adapted SF model derived from Birner et al. [8], a significant interaction between eccentricity and ONL-thickness (0.008 dB; *p* < 0.001), as well with EZ-thickness (−0.025 dB; *p* < 0.001) was observed. The volume of drusen and HRF had a negative association with PWS (−0.6 dB/nL; *p* = 0.007 and −9.56 dB/nL; *p* < 0.001). ONL thickness was associated with an increase in PWS with 0.060 dB/µm at an eccentricity of 5.2° (*p* < 0.001), while EZ thickness only showed a trend towards significance (0.017 dB/µm at an eccentricity of 5.2°; *p* = 0.1). The same was true for eccentricity (*p* = 0.087 at the mean values of ONLthickness and EZ thickness) (Table 3).

In the FF-model, eccentricity was negatively associated with PWS (−0.14 dB/°; *p* < 0.001), while no significant association between age and PWS was observed (*p* = 0.32). FD% also showed a negative association with PWS −0.005 dB/%; however, this effect was not statistically significant (*p* = 0.23) (Table 3).

In the third SFF model the EZ (0.04 dB/µm; *p* < 0.001) and ONL thickness (0.03 dB/µm; *p* < 0.001) had a significant positive association with PWS, while drusen volume and eccentricity showed negative associations (−1.69 dB/nL; *p* < 0.001 and −0.11 dB/°: *p* < 0.001, respectively). FD% on the other hand showed no significant association with PWS (*p* = 0.26). Also, in a univariable model, FD% did not show a significant association with PWS (−0.0008 [−0.011; 0.0094] dB/%; *p* = 0.88) (Table 3).

### 3.3. Model Comparison

AIC and BIC were lowest in the SF model (lower AIC and BIC indicate better model fit), whereas the FF model had the highest AIC and BIC values. When comparing the adjusted marginal R^2^ for explanatory power of the models the SF model explains the largest proportion of variance with a R^2^m = 0.32. The incorporation of flow information derived from OCT-A in addition to the structural OCT biomarkers slightly decreased model performance R^2^m = 0.29. Flow information without structural exhibited the least exploratory value with a R^2^m = 0.23. Note that the number of independent variables differs between models, which may affect the R^2^m, as it reflects variance explained by fixed effects (Table 4). ICCs, reflecting the variance explained by the random effect, ranged from 0.29 to 0.34.

## 4. Discussion

In this study, we compared exploratory predictive models integrating OCT-derived structural biomarkers, OCT-A-derived CC flow metrics, or a combination of both, to explain PWS measured by MP in eyes with iAMD. Across all multivariable analyses, structural biomarkers, specifically EZ thickness, ONL thickness, and drusen volume, emerged as the dominant predictors of retinal sensitivity. These findings are consistent with our previous work and with reports from other groups demonstrating strong structure–function relationships between outer retinal integrity assessed on structural OCT and visual function in AMD [8,9,43]. In contrast, CC flow information alone did not sufficiently predict retinal sensitivity, nor did the additional incorporation of CC FD% into a structural model improve explanatory performance. Model comparison using marginal R^2^, AIC, and BIC showed inferior performance of the flow-only model and a slight decrease in explanatory power when flow metrics were added to the structural model. These findings suggest that, at least in intermediate AMD and under photopic and mesopic testing conditions, CC FD% might not provide independent or additive information for predicting retinal sensitivity beyond established structural OCT biomarkers.

These results align with findings from the ALSTAR2 study, which investigated the relationship between CC perfusion loss and visual function [44]. In that cohort, no association was observed between global CC FD% and photopic or mesopic light sensitivity, whereas a strong relationship was demonstrated between CC FD% and rod-mediated dark adaptation (RMDA). The authors hypothesized that impaired metabolic exchange across the CC–Bruch’s membrane–RPE complex may compromise photoreceptor sustenance, particularly retinoid resupply, which is essential for dynamic rod function. Cones, in contrast, benefit from additional metabolic support through Müller glia and the retinal circulation [45], potentially preserving cone-mediated function during earlier disease stages. This differential vulnerability between rods and cones is consistent with established models of AMD progression [46]. Rod photoreceptors are thought to degenerate earlier than cones in AMD, a concept supported by observations that RMDA abnormalities often precede detectable changes in standard visual acuity or light sensitivity. Moreover, previous studies have shown that RMDA impairment is most pronounced in the parafoveal region (approximately 3–5° eccentricity) [47,48,49,50]. Curcio and colleagues proposed a center-surround model of photoreceptor resilience, in which foveal cones remain relatively protected during early disease stages, while parafoveal rods are particularly susceptible to metabolic stress and degeneration [46]. From this perspective, functional tests that preferentially probe rod-mediated parafoveal function may be more sensitive for detecting early vascular-driven dysfunction in AMD.

In the present study, retinal sensitivity was assessed using photopic and mesopic MP protocols, which predominantly reflect cone-mediated function. Consequently, the functional readouts employed may not have been optimally sensitive to CC-mediated metabolic impairment, potentially explaining the absence of a detectable flow-function association. This interpretation is further supported by prior work from Nassisi et al., who reported a significant correlation between reduced global CC perfusion and decreased scotopic mean retinal sensitivity [51]. Importantly, that study relied on averaged OCT-A images to quantify CC perfusion. Choriocapillaris perfusion is inherently pulsatile and subject to physiological variability related to systemic factors such as blood pressure [52], cardiac output [53], and physical activity [54]. As a result, single OCT-A acquisitions may be insufficient to capture subtle perfusion abnormalities, particularly in early disease stages. In healthy individuals, quantitative CC metrics have been shown to stabilize only after averaging four or more OCT-A acquisitions [55]. In the present study, CC analysis was based on a single OCT-A scan per eye, which may have limited sensitivity for detecting subtle flow alterations relevant to retinal sensitivity.

From a pathophysiological standpoint, CC impairment is believed to precede many structural manifestations of outer retinal degeneration. Histopathologic evidence suggests that complement-mediated membrane attack complex deposition initially damages subfoveal CC endothelial cells, leading to focal capillary loss. Drusen formation has been shown to occur preferentially in regions of CC loss [56], consistent with the observation that early AMD changes often originate in the central macula [57]. At this stage, central cone-mediated vision may remain relatively preserved due to alternative metabolic support pathways. As CC loss extends into the parafoveal region, where rods are more dependent on choroidal supply, functional deficits may first manifest as delayed RMDA rather than reduced photopic or mesopic sensitivity. In our cohort, structural sequelae of impaired CC perfusion, such as drusen accumulation, hyperreflective foci, and early photoreceptor disruption, were already present. Consequently, CC flow metrics may not have contributed additional independent information beyond what was already captured by structural OCT biomarkers. In this context, CC FD% may represent an upstream pathological process whose functional consequences are indirectly reflected through structural degeneration rather than through contemporaneous changes in cone-mediated retinal sensitivity.

Clinically, current approved AMD therapies predominantly target late-stage phenotypes, such as retinal fluid or geographic atrophy, when substantial vision loss has usually already occurred. There remains a critical need for quantifiable biomarkers capable of identifying high-risk patients and monitoring therapeutic effects during early, pre-atrophic disease stages. Due to the recent regulatory acceptance of EZ attenuation or loss as a clinical endpoint, it is already being used in clinical trials [58]. Its adoption as a trial endpoint represents an important step forward in this direction. Most EZ definitions using structural SD-OCT include the photoreceptor outer segment integrity [59], and its thickness can be regarded as a surrogate marker of photoreceptor metabolic health [60]. We hypothesize that future studies aiming to capture subtle point-wise flow–function relationships in early and intermediate AMD may need to combine rod-specific functional testing in the parafoveal region with averaged OCT-A measurements to mitigate physiological variability and enhance sensitivity.

The strengths of this study lie in the study design and validated DL-based segmentation of disease-specific biomarkers. We also used an innovative and previously validated method for CC FD% quantification. This method was previously performed in patients with geographic atrophy [31]. However, we demonstrate in this analysis that it is also applicable in iAMD eyes. Lastly, the precise overlay of not only MP and OCT, but also OCT-A, provides a comprehensive analysis of the complex interaction of macular structure, CC blood-flow, and retinal function. Nonetheless, several limitations need to be considered. Firstly, the study cohort is very small compared to other iAMD cohorts. Secondly, this was only a cross-sectional analysis, therefore no conclusions can be drawn regarding temporal course and progression. Thirdly, in these exploratory models, comparisons were assessed using only the AIC and BIC. These criteria provide information for relative model fit while penalizing complexity. However, they do not provide *p*-values or hypothesis tests. In addition, likelihood-based tests, cross-validation or validation with external data sets were not performed. This must be kept in mind when interpreting the results of the comparison. Furthermore, a major limitation of this study is the use of a single OCT-A acquisition per eye, despite the known variability of CC flow metrics [52,53,54]. This may have biased the performance of the models and reduced the ability to detect a potential contribution of CC FD%. In addition, PWS assessed by MP may predominantly reflect cone-mediated visual function, which could have influenced the observed structure–function relationships. Therefore, the conclusion that CC FD% has limited value should be interpreted with caution.

## 5. Conclusions

In conclusion we provide evidence that OCT-derived structural parameters such as drusen volume, EZ and ONL thickness, and HRF volume are the primary cross-sectional correlates of PWS. While blood-flow based metrics show limited baseline association, their clinical utility may reside in monitoring longitudinal progression rather than defining immediate structure–function relationships. Hence, these results should be regarded as hypothesis-generating rather than definitive. Further longitudinal studies with larger cohorts and a more robust OCT-A methodology are warranted to clarify the contribution of CC flow impairment in iAMD.

## Figures and Tables

**Table 1:** Descriptive statistics of point-wise sensitivity and point-wise OCT and OCT-angiography biomarkers stratified in device and run. Data are reported as mean ± SD if approximately normally distributed, and as median (Q1-Q3) otherwise.

Variable	MP-3 Run 1	MP-3 Run 2	Observations MP-3 Run 1/Run 2	MAIA2 Run 1	MAIA2 Run 2	Observations MAIA2 Run 1/Run 2
Sensitivity (dB)	25.88 ± 3.32	26.12 ± 3.39	850/807	22.49 ± 3.65	22.35 ± 3.63	820/820
EZ-thickness (µm)	28.89 ± 7.23	28.93 ± 7.41	850/807	28.72 ± 7.07	28.67 ± 7.17	820/820
ONL-thickness (µm)	64.64 ± 20.42	64.47 ± 20.43	850/807	63.57 ± 19.61	63.55 ± 19.53	820/820
Drusen volume (nL) ^1^	0.12 (0.01–0.45)	0.11 (0.01–0.47)	364/332	0.10 (0.02–0.45)	0.10 (0.02–0.47)	354/359
HRF volume (nL) ^1^	0.09 (0.07–0.15)	0.09 (0.07–0.16)	32/26	0.09 (0.07–0.21)	0.08 (0.07–0.22)	30/36
Flow-deficit percentage (%)	25.30 ± 12.30	25.27 ± 12.36	850/807	26.19 ± 12.71	25.42 ± 12.15	820/820

^1^ Drusen volume and HRF volume for values > 0.

**Table 2:** Spearman correlation of independent variables. EZ = ellipsoid zone, ONL = outer nuclear layer.

Variable	Age (Years)	EZ (µm)	ONL (µm)	Eccentricity (°)	Drusen Volume (nL)	Flow-Deficit Percentage
Age (years)	1.000	0.146	−0.087	−0.005	−0.115	0.093
EZ (µm)		1.000	0.085	0.237	−0.355	−0.006
ONL (µm)			1.000	−0.665	0.174	0.075
eccentricity (°)				1.000	−0.453	−0.214
Drusen volume (nL)					1.000	0.075
flow-deficit percentage (%)						1.000

**Table 3:** Multivariable mixed-effects models for (1) OCT-derived structure–function analysis. ONL and EZ are mean-centered; (2) OCT-A-derived flow–function analysis; and (3) OCT and OCT-A combined structure–flow–function analysis. OCT = optical coherence tomography; OCT-A = OCT-angiography; CI = confidence intervals; R^2^m = marginal R^2^. HRF = hyperreflective foci; ONL = outer nuclear layer; EZ = ellipsoid zone; R° = eccentricity; nL = nanoliters; (*) please note that the analyses are exploratory, hence the interpretation of the *p* values is descriptive.

OCT–Structure–Function Correlation				
Variable	Estimate	Lower 95% CI	Upper 95% CI	*p* Value *
MAIA2 vs. MP-3 device	−3.556	−3.743	−3.370	<0.0001
Drusen volume (nL)	−0.602	−1.039	−0.165	0.007
HRF volume	−9.561	−12.860	−6.262	<0.0001
ONL (µm, at R = 5.2°)	0.060	0.049	0.072	<0.0001
EZ (µm, at R = 5.2°)	0.017	−0.003	0.038	0.1
R° (at PR = 28.8 µm, ONL = 64.1 µm)	0.060	−0.009	0.128	0.087
ONL: R	0.008	0.006	0.010	<0.0001
EZ: R	−0.025	−0.031	−0.019	<0.0001
**OCTA–Flow–Function Correlation**				
MAIA2	−3.633	−3.834	−3.432	<0.0001
Age (years)	−0.066	−0.201	0.070	0.32
Eccentricity (°)	−0.139	−0.175	−0.103	<0.0001
Flow deficit percentage (%)	−0.005	−0.014	0.003	0.23
**Structure–Flow–Function Correlation**				
MAIA2	−3.585	−3.776	−3.394	<0.0001
Age (years)	−0.077	−0.218	0.065	0.27
EZ (µm)	0.044	0.023	0.064	<0.0001
ONL (µm)	0.026	0.017	0.036	<0.0001
Eccentricity (°)	−0.112	−0.175	−0.048	0.00062
Drusen volume (nL)	−1.687	−2.089	−1.286	<0.0001
Flow deficit percentage (%)	0.005	−0.004	0.013	0.26

**Table 4:** Comparison of model performance according to the Akaike information criterion (AIC), Bayesian information criterion (BIC), the marginal R^2^ (R^2^_m_) and intraclass correlation coefficients (ICC).

Model Type	AIC	BIC	R^2^_m_	ICC
OCT–structure–function (SF) correlation	16,048.47	16,115.58	0.32	0.33
OCT-A–flow–function (FF) correlation	16,554.52	16,597.23	0.23	0.29
Structure–flow–function (SFF) correlation	16,205.18	16,266.19	0.29	0.34

## Data Availability

The datasets presented in this article are not readily available because the data are part of an ongoing study. Requests to access the datasets should be directed to gregor.reiter@meduniwien.ac.at.

## Supplementary Materials

The following supporting information can be downloaded at: https://www.mdpi.com/article/10.3390/jcm15093392/s1, Table S1. Variance inflation factors (VIF) of each model. In the structure-function (SF)-model, outer nuclear layer (ONL), ellipsoid zone (EZ) and eccentricity are mean centered. HRF = hyperreflective foci. Figure S1. Residual vs. fitted plot for the structure-function (SF) model. Figure S2. Residual vs. fitted plot for the flow-function (FF) model. Figure S3: Residual vs. fitted plot for the structure-flow-function (SFF) model.

## Abbreviations

The following abbreviations are used in this manuscript:

AIC	Akaike information criterion
AMD	age-related macular degeneration
ART	automated real time
BCVA	best-corrected visual acuity
BIC	Bayesian information criterion
BM	Bruch’s membrane
CC	choriocapillaris
CFP	color fundus photography
cRORA	complete RPE and outer retinal atrophy
ELM	external limiting membrane
FD%	flow deficit percentage
FF	flow–function
IRF	intraretinal fluid
ICC	intraclass correlation coefficient
SRF	subretinal fluid
LLVA	low-luminance visual acuity
MNV	macular neovascularisation
MP	microperimetry
NIR	near infrared reflectance image
OB-RPE	outer border of the RPE
OCT	optical coherence tomography
OCT-A	OCT-A
ONL	outer nuclear layer
OPL	outer plexiform layer
IZ	interdigitation zone
PED	pigmentary epithelium detachment
HRF	hyperreflective
PWS	point-wise retinal sensitivity
REML	reduced maximum likelihood
RMDA	rod-mediated dark adaption
ROI	region of interest
RPE	retinal pigment epithelium
RS	retinal sensitivity
SDD	subretinal drusenoid deposits
SF	structure–function
SFF	structure–flow–function
SIFT	Scale-Invariant Feature Transform
SLO	scanning laser ophthalmoscopy
VIF	variance inflation factor
