# Supplementary material for: Choriocapillaris Flow-Enriched Prediction of Retinal Sensitivity Using OCT-Derived Biomarkers in Intermediate Age-Related Macular Degeneration
Source: J Clin Med. 2026 Apr 29;15(9):3392. doi: 10.3390/jcm15093392 (PMC13163744; doi:10.3390/jcm15093392)

Supplemental Material

Supplement table S1. Variance inflation factors (VIF) of each model. In the structure-function (SF)-model, outer nuclear layer (ONL), ellipsoid zone (EZ) and eccentricity are mean centered. HRF = hyperreflective foci.

**Term**	**VIF**
**SF**	
MAIA2 vs. MP-3 device	1.01
Drusen volume (nL)	2.81
HRF volume	1.27
ONL (µm)	4.92
EZ (µm)	1.90
Eccentricity (°)	4.56
ONL: Eccentricity	1.70
EZ: Eccentricity	1.53
**FF**	
MAIA2 vs. MP-3 device	1.00
Age (years)	1.00
Eccentricity (°)	1.07
Flow deficit percentage (%)	1.07
**SFF**	
MAIA2 vs. MP-3 device	1.01
Age (years)	1.00
EZ (µm)	1.82
ONL (µm)	3.18
Eccentricity (°)	3.76
Drusen Volume (nL)	2.27
Flow deficit percentage	1.09

Supplement Figure S1: Residual vs. fitted plot for the structure-function (SF) model

SF

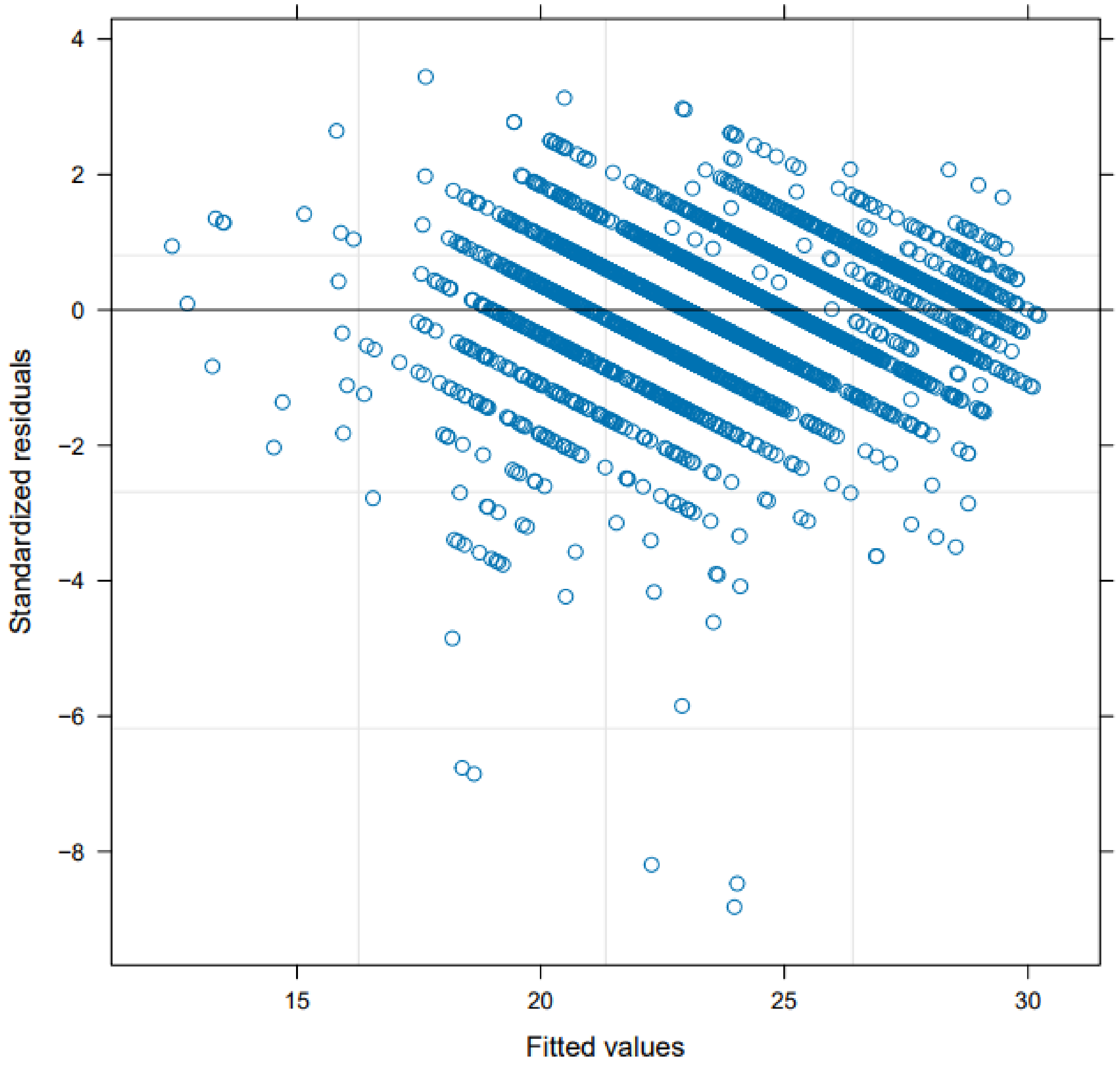

Supplement Figure S2: Residual vs. fitted plot for the flow-function (FF) model

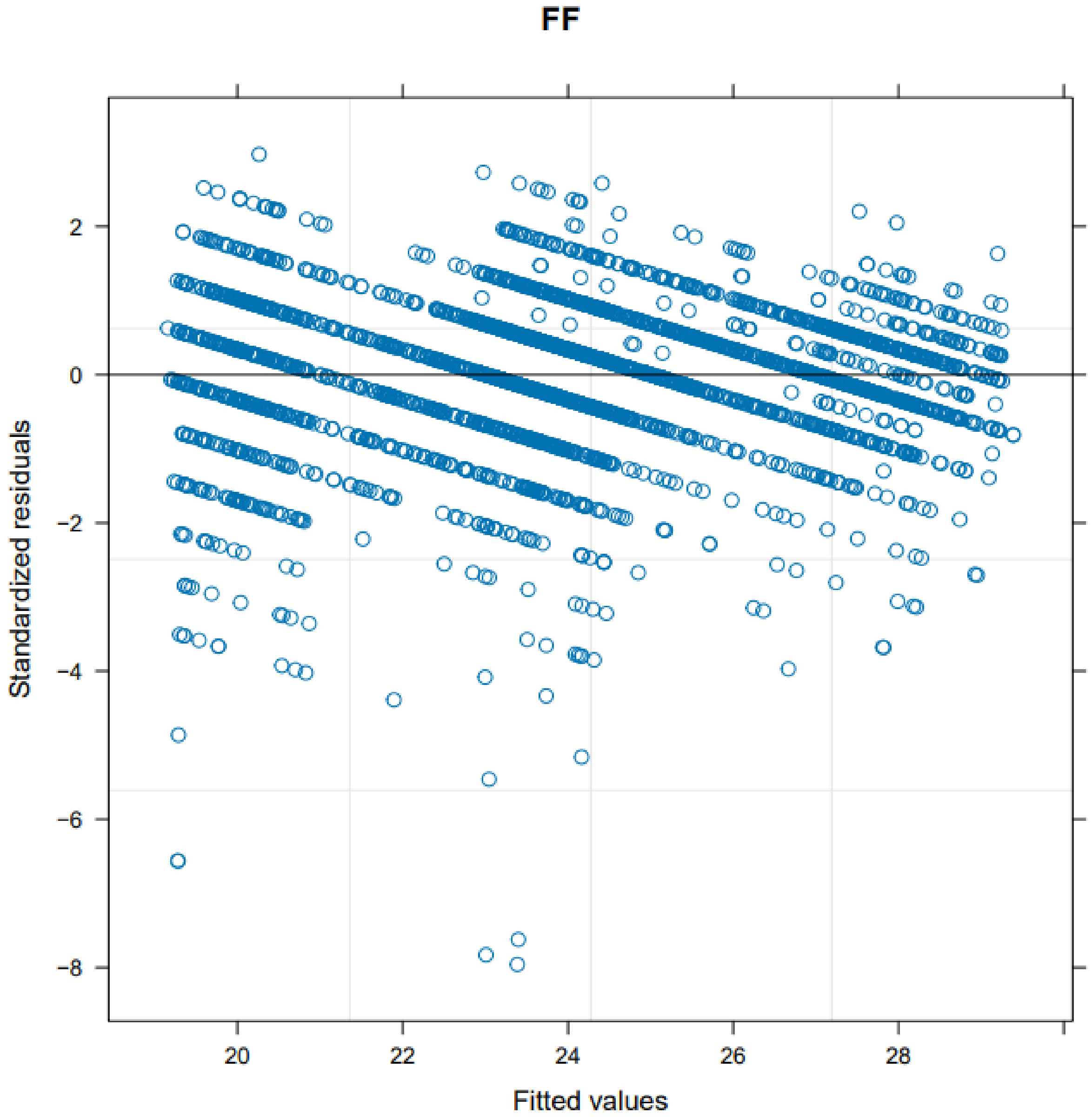

Supplement Figure S3: Residual vs. fitted plot for the structure-flow-function (SFF) model

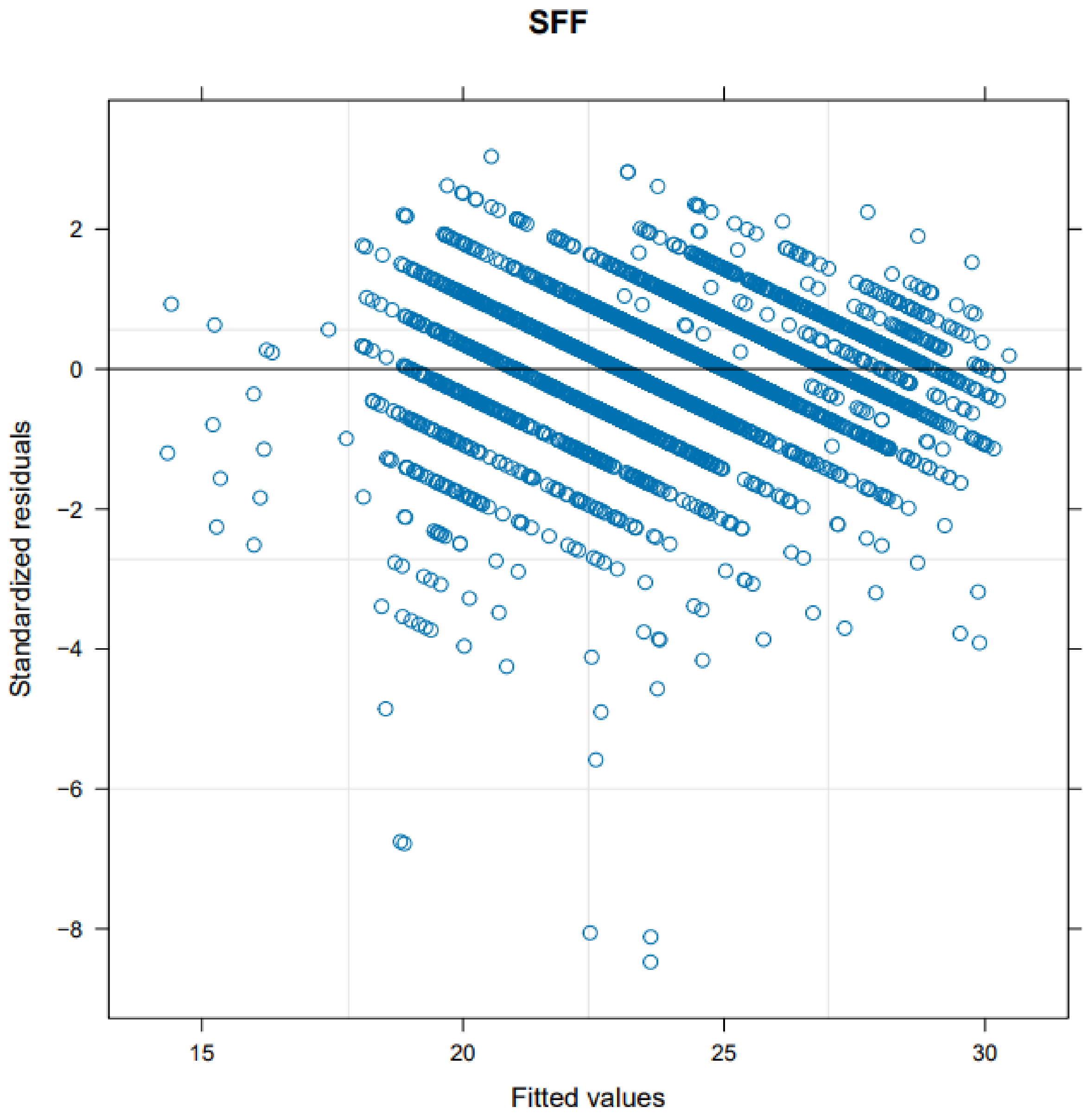

Supplement: Supplementary file 1 [file jcm-15-03392-s001.zip › jcm-4236850-supplementary.pdf]
